# Sodium Gill Potential as a Tool to Monitor Valve Closure Behavior in Freshwater Clam *Corbicula fluminea* in Response to Copper

**DOI:** 10.3390/s8095250

**Published:** 2008-09-01

**Authors:** Chung-Min Liao, Chieh-Ming Lin, Li-John Jou, Wei-Yu Chen

**Affiliations:** 1 Department of Bioenvironmental Systems Engineering, National Taiwan University, Taipei, Taiwan 10617, R.O. China; E-Mails: cmliao@ntu.edu.tw; r94622016@ntu.edu.tw; f96622016@ntu.edu.tw; 2 Department of Biomechatronic Engineering, National Ilan University, Ilan, Taiwan 260, R.O. China; E-mail: ljjou@niu.edu.tw

**Keywords:** Clam, *Corbicula fluminea*, Bioavailability, Gill membrane potential, Electrophysiology, Valve closure behavior

## Abstract

Valve closure behavior in freshwater clam *Corbicula fluminea* is a biologically sensitive endpoint. The purpose of this paper was to derive an electrophysiological response model of *C. fluminea* to assess copper (Cu)–sodium (Na) interactions in gill membrane, whereby valve closure behavior and Cu toxicity could be monitored. The proposed model was based on the integration of Cu bioavailability, Na and Cu internalizations, and electrochemically-based gill potentials. Based on Na active transport under non-equilibrium conditions, predicted gill potential of −8.2 mV agreed reasonably well with published the measured transepithelial potential of −7 mV in *C. fluminea*. Our proposed framework captured the general features observed in model applications including: (*i*) 50% inhibitory Cu^2+^ activities for Na membrane potential (*E*_Na_) and uptake rate (*J*_Na_) were estimated to be 0.072 and 0.043 μM, respectively, with a stoichiometry of 3Cu^2+^: 1*E*_Na_ and 1*J*_Na_; (*ii*) the external Cu^2+^–dependent internal Na concentration could be parsimoniously estimated, and (*iii*) the site-specific clam gill potentials could be monitored. Here we provided a new approach to monitor waterborne metal toxicity to reduce the nationwide economic losses due to bans on harvesting of contaminated clam and the potential risks to the health of clams.

## 1. Introduction

**Figure 1.**
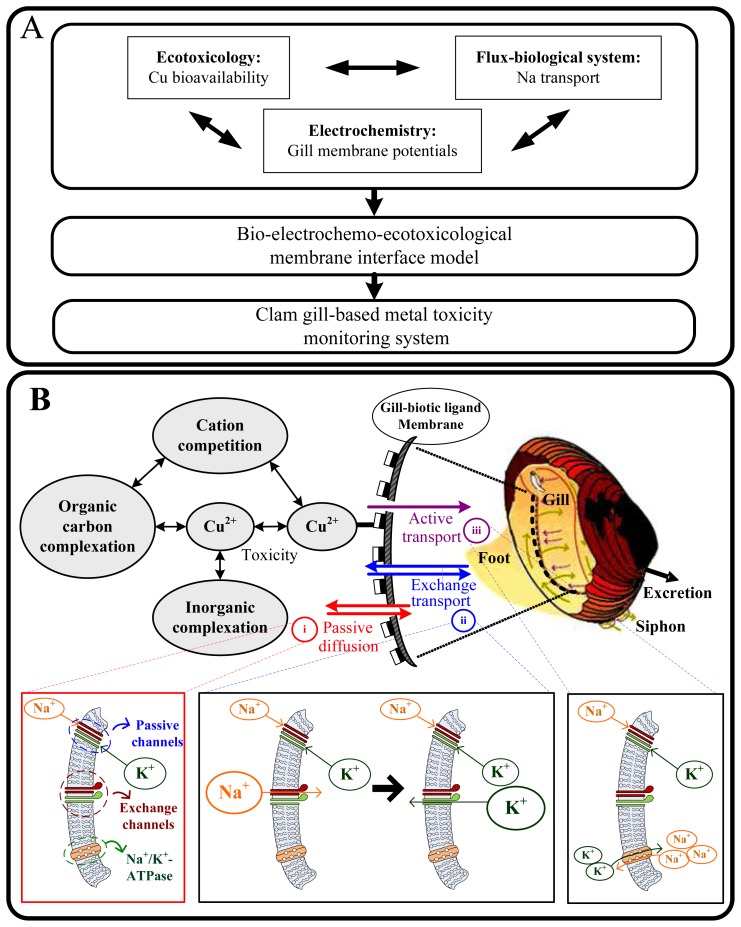
**(A)** Schematic of our proposed framework inspired from key concepts of ecotoxicology, biological physiology, and electrochemistry to derive a clam gill-based membrane interface model for the future design of environmental biomonitoring and prediction of metal toxicity. **(B)** BLM-based Cu bioavailability associated with the affinity and capacity of gill to bind copper based on site-specific water quality parameters in that physiological mechanisms of Na transport in gill-biotic ligand membrane including (*i*) passive diffusion, (*ii*) exchange transport, and (*iii*) active transport.

Freshwater clam *Corbicula fluminea* is a commercially important native bivalve species and has a high market value to Taiwan's aquaculture (http://www.fa.gov.tw), with wide farming distribution in the western and eastern coastal areas of Taiwan. We recognized that bivalves were a popular choice of sentinel organisms for biological early warning system (BEWS) to monitor the impact of pollutants in aquatic ecosystems [1-10]. Moreover, the dynamic metal speciation analysis in aquatic ecosystems is emerging as a powerful tool basis for prediction developments of bioavailability and reliable risk assessment strategies [11-13]. Here we proposed a framework inspired from key concepts of ecotoxicology (i.e., Cu bioavailability), biology (i.e., Na^+^ transport kinetics and Na^+^/K^+^–ATPase activity) and electrochemistry (i.e., gill potentials) to present the practical implications of this integrated knowledge based on a rigorous quantitative methodology (Figure 1A).

Morgan and Wood [14] and Zhou *et al.* [15] indicated that the key mechanism of metal toxicity consists of reduction in Na^+^ uptake by blockade of Na^+^/K^+^–ATPase in the gill epithelia of freshwater rainbow trout (*Oncorhynchus mykiss*). Na^+^/K^+^–ATPase activity has been reported in gills of the oyster (*Crassostrea virginica*), hard clam (*Mercenaria mercenaria*), and freshwater mussel (*Carunculina texasensis*) [16, 17]. Generally, Na^+^/K^+^–ATPase pumps generate concentration gradients of cations across membranes in nearly all cells, providing a polar transmembrane pathway. In each transport cycle, up to a hundred times a second, a single Na^+^/K^+^–ATPase pump exchanges three cytoplasmic Na^+^ ions for two extracellular K^+^ ions and hydrolyses one molecule of ATP, involving an active transport mechanism [18, 19].

Organisms do not have specific transport systems for the vast majority of compounds that are internalized by the cell. Thus, most compounds must borrow existing pathways designed for the essential elements: transport through ion channels, carrier-mediated transport, and active transport, where ions are moved against electrochemical gradients driven by the free energy of ATP hydrolysis. It is known that most trace metals are moved down their electrochemical gradients by simple diffusion (passive transport), diffusion through ion channels or by facilitated diffusion (exchange transport). Once inside the cell, transition metals often play important roles as coenzymes or participate in catalytic processes, due to their ability to adopt several different redox states [20].

In gills of marine teleosts and freshwater bivalves, the Na^+^ transport system is thought to involve transmembrane pores, through which Na^+^ ions move down an electrochemical activity gradient. It indicates that most of the Na flux-dependent gill potentials occur through the active transport mechanism(s) [21-25]. Many studies have been reported that ion transport processes in freshwater bivalves exhibit saturation kinetics [24, 26-29].

McCorkle and Dietz [24] indicated that Na transport in *C. fluminea* is efficient and Na balance could be examined by partitioning Na flux into three processes: (*i*) passive diffusion (efflux = diffusion + excretion = 2.87 ± 0.76 μM Na g^-1^ dw h^-1^ and influx = 0.50 μM Na g^-1^ dw h^-1^), (*ii*) exchange diffusion (influx = efflux = 5.91 ± 0.80 μM Na g^-1^ dw h^-1^), and (*iii*) active transport (influx = 2.41 μM Na g^-1^ dw h^-1^) (Figure 1B).

The transepithelial potential (TEP) that is necessary to maintain Na^+^ electrochemical equilibrium can be estimated by the Nernst equation [24]. McCorkle and Dietz [24] reported that the estimated Nernst TEP of −74 mV dose not equal to the measured TEP of −7 mV, suggesting active transport in *C. fluminea*. Nernst equation can be used to describe the relationship between electrical potential (*E*_m_) across a membrane and the ratio of the concentrations (*C*_i_/*C*_o_) and valences of ions on either side of the membrane. Nernst potential of Na^+^ is one of the present key concepts and has many applications in biological systems [30].

The purpose of this paper is to provide a bio-electrochemically inspired framework by incorporating bioavailability and flux transport kinetics into an electrochemical model. The approach facilitates an electrophysiological response model that describing Cu-Na interactions in clam gill membrane for the prediction of metal toxicity and future design of biomonitoring system in aquaculture settings. Hopefully, our preliminary initiative can provide a precautionary monitoring programme for assessing the environmental impact of waterborne metals to freshwater species. Thus the economic losses nation-widely can be reduced from bans on harvesting of contaminated clam. Moreover, the potential risks on the health of clams and people who intake the contaminated clam can also be reduced.

## 2. Results and Discussion

### 2.1 Model performances

The gill membrane potential (Nernst potential) necessary to maintain Na in electrochemical equilibrium is predicted to be −84.2 (95% CI: −93.9 to −67.9) mV; this modeled value is comparable to the estimated value of –74 mV by [24] (Table 1). Our calculated gill potential in non-equilibrium conditions of −8.2 mV based on active transport of Na is reasonably agreed with the measured transepithelial potentials of −7 mV by [24] (Table 1). This result indicates that active transport of Na can be used to account for the gill potential of clam when valves are open and the siphoning activity is engaged.

**Figure 2.**
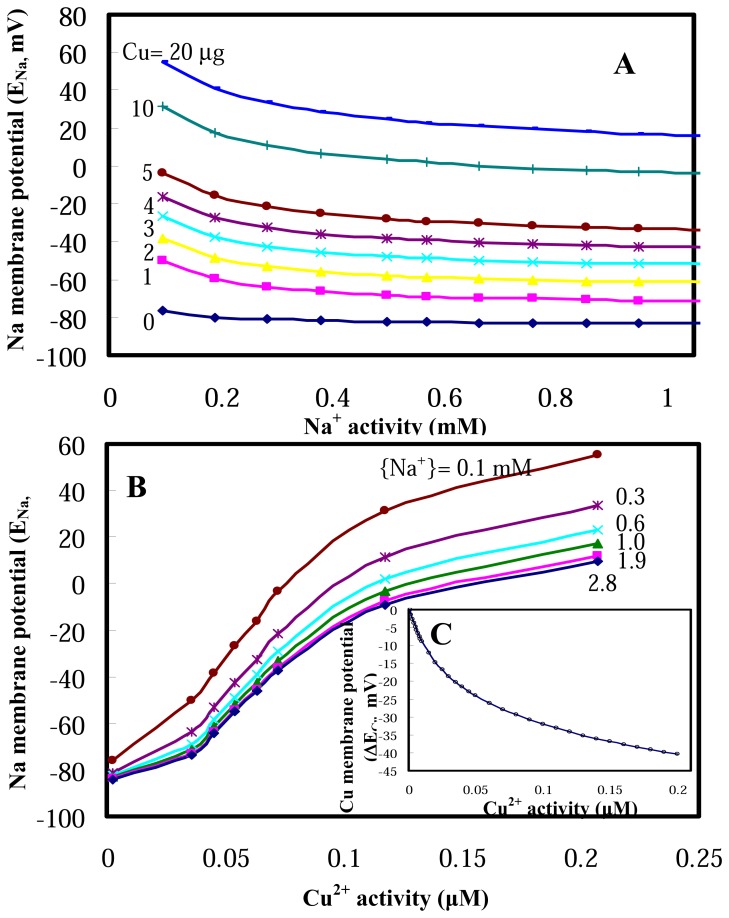
Predictions of clam gill membrane potentials. **(A)** Na^+^ activity-dependent Na membrane potentials in response to Cu ranging from 0 to 20 μg L^-1^. **(B)** Cu^2+^ activity-dependent Na membrane potentials at Na^+^ activity ranging from 0.1 to 2.8 mM. **(C)** Cu membrane potential changes range from 0 to −40.4 mV varied with Cu^+^ activity ranging from 0–0.2 μM.

Na^+^ activity–dependent Na membrane potentials increase from negative to positive with increasing Cu concentrations, whereas Cu^2+^ activity–dependent Na membrane potentials increase from negative to positive with decreasing Na^+^ activities (Figures 2A, B). Figure 2A depicts that gill potentials are depolarized from controlled −84 mV to +16 mV in response to waterborne Cu increasing from 0 to 20 mg L^-1^. Figure 2B reveals that when Cu^2+^ activities increase from 0 to 0.2 μM, a depolarization process drives the gill potentials from controlled −84.2 mV to nearly 55 mV and 10 mV at Na^+^ activities of 0.1 and 2.8 mM, respectively. On the other hand, Cu membrane potential changes decrease with increasing of Cu^2+^ activities (Figure 2C).

**Figure 3.**
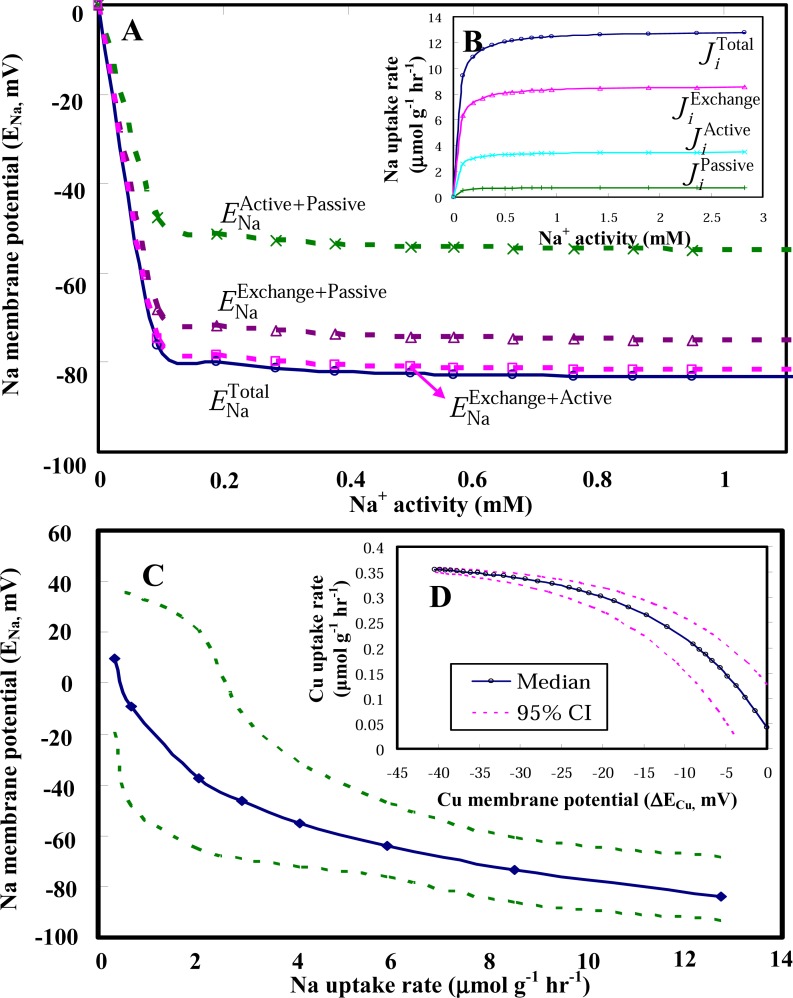
Physiological and electrophysiological kinetics of Na flux partitions: **(A)** Na membrane potentials and **(B)** the unidirectional Na influx. Predicted the profiles of ion uptake rate – Nernst membrane potentials for **(C)** Na and **(D)** Cu.

The predicted Na^+^ activity–dependent transport process-specific Na membrane Nernst potentials decrease sharply when Na^+^ activities are less than 0.1 mM and stay nearly constant when Na^+^ activities are larger than 0.1 mM (Figure 3A). The partitioning ratios of the unidirectional influx of Na in *C. fluminea* to the total influx are based on the empirical data from [24] (Figure 3B). The predicted Na uptake rate-Nernst membrane potential profile indicates that Na membrane potentials decrease from +10 to −84 mV with increasing Na uptake rates ranging from 0.1 – 13 μmol g^-1^ h^-1^ (Figure 3C). Decreasing of Cu uptake rates from 0.35 − 0.05 μmol g^-1^ h^-1^ results in a increasing Cu membrane potential changes from −40 − 0 mV (Figure 3D).

**Figure 4.**
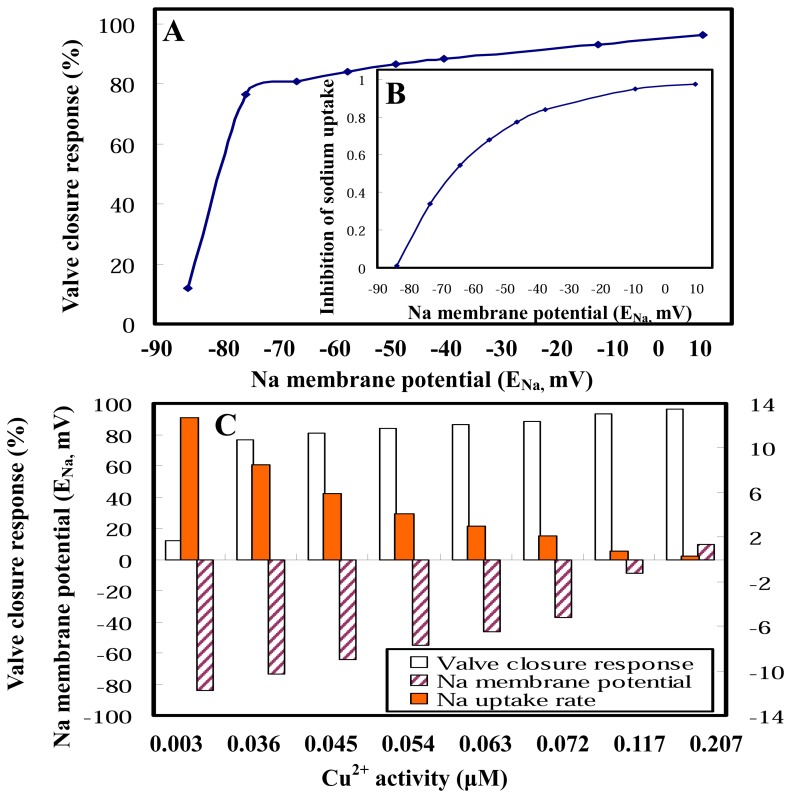
Predicted biological and electrophysiological responses that includes clam valve closure behavior, Na uptake, and gill potentials. **(A)** Relationships between closure response and electrophysiological properties (Na membrane potential). **(B)** Relationships between inhibition of Na uptake and Na membrane potential. **(C)** Cu^2+^ activity-dependent interactions, showing that changes of valve closure response, Na uptake rate, and Na membrane potential.

We predicted the relationship between clam valve closure behavior and electrophysiological properties by using the valve closure response–Na membrane potential profile to assess the biological responses (Figure 4A). A sharp change of valve closure responses from 10 to 76% occurred when Na membrane potentials increase from −84 to −74 mV. Clams experience a smooth closure response from 76 to 100% when Na membrane potentials notably increase from −74 to 10 mV (Figure 4A). The 50% inhibitory Na membrane potentials (IP50) for valve closure response and Na uptake rate are, respectively, −73.54 mV and −64.16 mV (Figures 4A, B). Figure 4C demonstrates the Cu^2+^ activity–dependent interplay among valve closure response behavior, gill potentials, and Na uptake rates, revealing a substantial link between ecotoxicology (Cu bioavailability) and electrophysiology (Na transport and gill potentials) in *C. fluminea*. It plays a crucial role in determining the kinetics of gill ligand binding mechanisms.

### 2.2 Model applications

**Figure 5.**
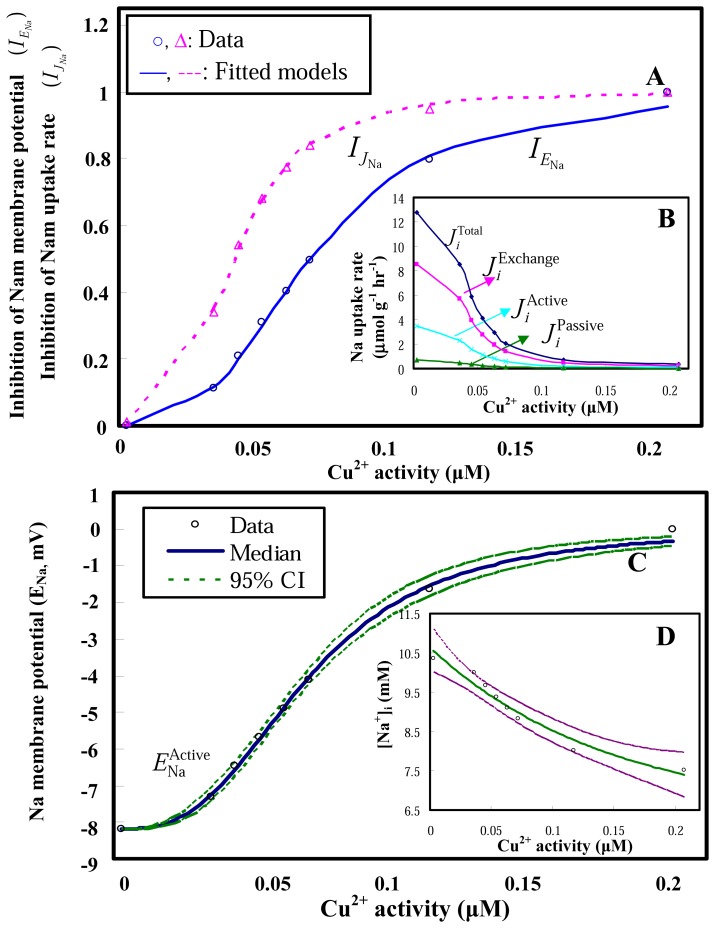
Predicted dose-response profiles. **(A)** Hill-based dose-response profiles showing the relationships between inhibitions of Na membrane potential/Na uptake rate and Cu^2+^ activity. **(B)** Cu^2+^ activity-dependent Na uptake rate profiles partitioning with different transport mechanisms of exchange, passive, active, and total influxes. **(C)** Prediction of active gill potentials at a non-equilibrium condition, showing increasing of Cu^2+^ activities has a mild depolarization process from −8.2 to 0 mV. **(D)** Predicted Cu^2+^ activity-dependent internal Na concentration in blood showing the changes of internal Na concentration from 10.56 to 7.40 mM varied with Cu^+^ activities ranging from 0 – 0.21 μM.

We reconstructed Hill-based dose-response relationships between inhibitions of Na membrane potential and Na uptake rate and Cu^2+^ activities. The results indicate that 50% inhibitory concentrations (IC50) for Na membrane potential and uptake rate are best estimated (*r*^2^ = 0.99) to be 0.072 and 0.043 μM Cu^2+^with fitted Hill coefficients of 2.95 and 3.29, respectively (Figure 5A). The Cu^2+^activity-dependent Na uptake rate profiles partitioning with different transport mechanisms are shown in Figure 5B. To assess the effect of external Cu^2+^ activity on gill potential at nonequilibrium conditions, we estimated the Cu^2+^ activity-dependence gill active potentials based on the Cu^2+^ activity-dependence Na uptake rate profiles (Figure 5B).

At a nonequilibrium condition, the predicted active gill potentials have a mild depolarization process from −8.2 to 0 mV when external Cu^2+^ activities increase from 0 – 0.2 μM (Figure 5C). The result demonstrates that a sigmoidal dependence on the external Cu^2+^ activities followed a best fitted Hill equation $E_{\text{Na}}^{\text{Active}}=-8.2+\left(\left(E_{\max}\times {\left\{{\text{Cu}}^{2+}\right\}}^{n})/{\left({\text{EC}}_{\text{active}}50\right)}^{n}+{\left\{{\text{Cu}}^{2+}\right\}}^{n}\right)\right)$ with a Hill coefficient *n* = 2.97, *E*_max_ = 8.2, and the effective Cu activity that block 50% of active Na^+^ channel transport (EC_active_50) is estimated to be 0.072 μM (*r*^2^ = 0.99).

This result implies that three Cu^2+^ ions bind to a single site in the outer gill membrane pore of the Na^+^ channel to block the active Na transport. It results in a depolarization-induced shift of clam behaviors such as daily valve closing/opening rhythm and siphoning capacity from high to low in response to waterborne Cu. We therefore incorporated the fitted $E_{\text{Na}}^{\text{Active}}$ function into Eq. (18) to estimate internal Na concentration in *C. fluminea*. A parsimonious exponential function ([Na^+^]_i_ = 6.37+4.27×exp ({Cu^2+^}/0.147), *r*^2^ = 0.96) best describes the relationship between the external Cu^2+^ activity and internal Na concentration in blood (Figure 5D).

**Figure 6.**
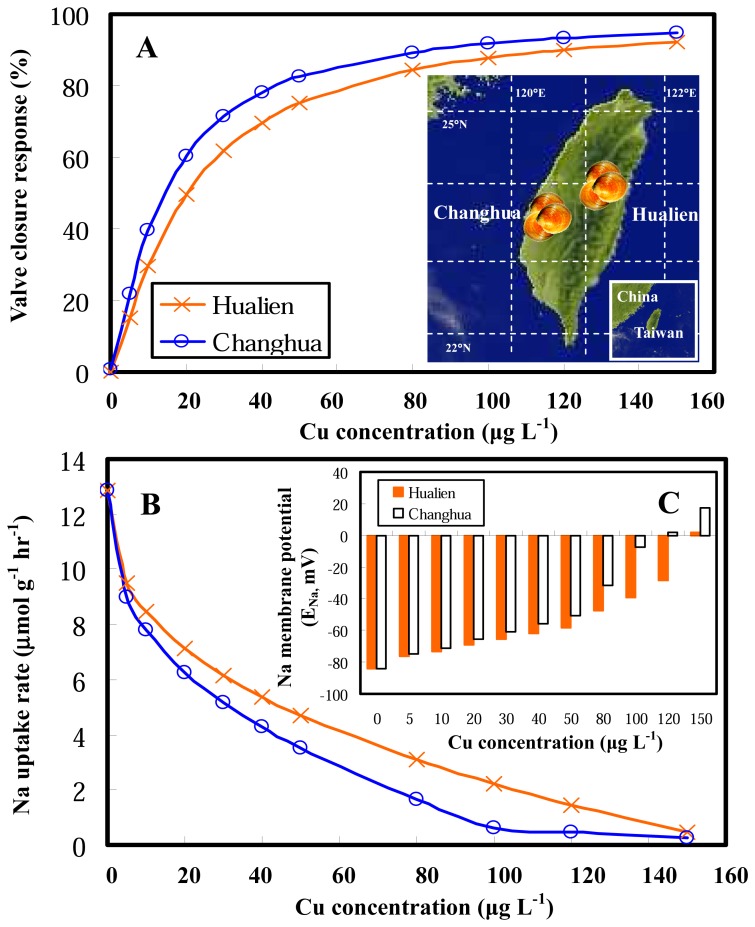
Simulations of freshwater clam *C. fluminea* physiological and electrophysiological characteristics in response to waterborne Cu for major clam farms located at Changhua and Hualien, showing the site-specific toxicity effect of **(A)** valve closure response, **(B)** Na uptake rates, and **(C)** Na membrane potentials varied with Cu concentrations ranging from 0 – 150 μg L^-1^.

We employed our proposed framework to predict site-specific clam valve closure behavior (Eq. (T1)), Na uptake rate (Eq. (T7)), and associated gill membrane potentials in response to waterborne Cu (Eq. (7)) for major clam farms located at Changhua and Hualien, respectively, in the southwestern and eastern Taiwan. The adopted water quality data for Changhua and Hualien farms are listed in Table 2. Figure 6 reveals that physiological and electrophysiological characteristics of *C. fluminea* in Changhua clam farm are more sensitive than those in Hualien. This result suggests that the proposed bio-electrophysiological model could be used to test the bivalve biological and electrophysiological response abilities to close its shell as an alarm signal to reflect clam's health when exposed to waterborne metals by taking account of the metal bioavailability. Figure 6C also shows that the site-specific clam gill potentials could be predicted by the present model and could also be used in the assessment of metal ion bioavailability in the aquaculture settings.

### 2.3 Clam gill potential as a determinant in environmental risk assessment

We proposed the mechanistic models based on concepts obtained from BLM, M-M kinetics, and electrochemistry to explore the interplay among valve closure behavior, Na/Cu transport, and gill membrane potentials.

The present model can be used to describe the electrophysiological response in *C. fluminea* in response to waterborne Cu. A *Corbicula*-based electrophysiological response framework is developed by incorporating previous Cu-BLM-*Corbicula* model and flux-biological response kinetics into an electrochemical mechanism for providing an accurately measuring endpoint based on a clam-gill membrane interface. We estimated acute Cu toxicity that is associated with inhibition of Na^+^ uptake rate involving active Na^+^ membrane potential at the gill interface.

Electrophysiological-based biological responses have been widely applied to variety of species for investigating internal physiological mechanism-based toxicity effects [21, 25, 32, 33] suggested that Na^+^ transmembrane potential difference in mussels was a reasonably good indicator of toxicity. Furthermore, our approach should have a potential to estimate gill damage and individual death by inhibition of Na^+^/K^+^–ATPase activity-induce electrophysiological potentials [32].

The estimated dose-response profiles in Figure 5 are the pivotal results for environmental risk assessment in this paper. From our analysis, we predicted the effective Cu activity that blocks 50% of active Na^+^ channel transport (EC_Active_50) is 0.072 μM with a stoichiometry of $3{\text{Cu}}^{2+}:1E_{\text{Na}}^{\text{Active}}$. That causes a depolarization of the gill membrane by external Cu ion activity to result in a significant decrease in active Na influx that directly/indirectly disturbs the clam valve daily opening/closing rhythm. Our results also implicate that the uptake and toxicity of Cu is much better correlated with activity at gill membrane surface than with activity in the bulk phase medium.

Kinraide [33] argued that the BLM incorporated with free ion activity model (FIAM) generally do not take into account the membrane potentials, although the BLM might consider the gill surface. Consequently, it is often inadequate for the expression of ionic effects, suggesting that membrane potential concept could be used as a general index in assessment of the bioavailability of ions. Kinraide [33] further pointed out that the BLM involves competition among ions as the mechanism of interaction. Site-specific competition, however, cannot explain some instances of interaction. Diffuse electrostatic effects appear to account for the interactions entirely because ions of opposite charge are unlikely to compete for ligand binding sites.

Here we suggested that although site-specific competition among ions might occur, competition only cannot be assessed without consideration of membrane potentials. Therefore we recommended that the effects of membrane potential on the gill-biotic ligand could be incorporated with the effects of binding site competition to assess the metal toxicity. Thus the bioavailability of a metal ion in solution might be dominated more by the membrane potential-depolarizing processes of ions than by competitive interactions of the ions at a ligand binding site.

Cereijido et al. [34] pointed out that epithelia unambiguously demonstrated active Na transport that was first observed by Ussing and Zerahn [35], showing that frog skin can actually transport a net amount of Na^+^ in the inward direction and in the absence of an external electrochemical potential gradient. Assessments of metal risk have been specific for environment and organism. In these cases, our framework that relates the biotic response to Na active transport-induced active membrane potentials might be adequate. Gill membrane depolarization processes do occur in aquatic animals in response to external stressors [36, 37].

Membrane surface activities associated with site-specific binding and competition should be incorporated into the BLM as proxies to represent the bulk phase concentrations where the gill membrane surface activities must be computed from active Na potentials [22, 23, 25, 38, 39]. Bricelj et al. [37] integrated behavioral, electrophysiological, and molecular biological approaches to study the Na channel mutation that leading to saxitoxin resistance in clams. They indicated that the increased accumulation of toxin in resistance clams points to this resistance mutation as an important risk factor for human paralytic shellfish poisoning (PSP) resulting from the consumption of this species.

Hence our proposed framework linking Cu bioavailability and electrophysiological responses of *C*. fluminea could provide a practical environmental risk assessment tool. We further suggests that clam gill membrane potential could be adapted as an electrophysiological endpoint of bioavailability and metal toxicity action used in environmental risk analysis to enhance broad risk management strategies [37, 40].

Merging the concepts of ion bioavailability and internalization flux, such as BLM, and M-M kinetics, with the gill membrane potentials described by Nernst and Ussing flux ration equations may provoke new measurement and modeling approaches for monitoring the behavioral dynamics of freshwater bivalves. A new way forward would be a further effort to distinguish between inherent kinetic properties of individual clams and the suite of environmental constraints to response that frequently exists *in situ*. Although further experiments to investigate the details of multiple transports in biological membranes are underway, the results described here demonstrate that the integration of Cu bioavailability and electrophysiological responses of *C. fluminea* provided a means to reconfigure mechanisms of active transport across epithelia in bivalves.

The model can be readily extended to account for additional phenomena, such as ATPase activity and NaCl uptake in the gills of freshwater bivalves. The Nernst equation presented in this paper might be linked with Goldman-Hodgkin-Katz equation, *V*_r_ = *RT*/*F*ln{(*P*_Na_/*P*_Cl_[Na^+^]_o_ + [Cl^−^]_i_ / *P*_Na_ / *P*_Cl_[Na^+^]_i_ + [Cl^−^]_o_)}where the subscripts *o* and *i* indicate external and internal ion activities to values of *V*_r_ as a function of Na^+^ and Cl^−^ activities, to calculate resting membrane potential (*V*_r_) and to estimate the permeability ratio of *P*_Na_/*P*_Cl_ while Cl^−^ ion transport in *C. fluminea* is considered. The model has the additional feature that it can be used to address one of the key challenges in biological membrane kinetics, namely, how to determine the active gill potentials of a living clam that responds to external Cu concentrations. Because the model captures the reorganization of biological and electrophysiological characteristics of clam in response to external free metal ion activities, it can be used as a framework to design and interpret appropriate experiments.

### 2.4 Implications for biomonitoring systems

Our results may have practical implications for future technological and biomonitoring applications. These results provide a scientific basis for future designing the environmental biomonitoring systems. Cu bioavailability, physiological mechanism of Na transport, and electrochemical transmembrane that has an important theoretical advantage over traditional toxicity models [41,42] to potentially take into account of both clam physiological and environmental factors affecting metal-induced biological responses. Practically, we have to first observe the valve daily rhythm dynamic fashion in response to Cu to indirectly obtain a BLM-based concentration-time-response profile. In the following step, we need to estimate the waterborne free Cu^2+^-activity {Cu^2+^} by using the major physiological parameters in *C. fluminea* and thus that a real waterborne Cu ion concentration [Cu^2+^] can then be evaluated depending on the site-specific water quality conditions. We focus on calcium, magnesium, and sodium because they have positive effects against copper toxicity based on BLM scheme [43]. The possible toxicity of copper hydroxide complexes would imply that at the higher pH, less would be needed to exert the same toxic effect. The temperature also has significant effects on the biological behavior or chemical speciation of a toxicant as well. In the future work, such a biomonitoring tool will be implemented to detect toxic effects of multiple metals.

Our proposed model can be applied to develop an artificial clam gill-based membrane interface that mimics ion transports of Na and Cu in *C. fluminea* to evaluate the relationships between gill potentials and Na and Cu internalization fluxes. The Na^+^/K^+^–ATPase activity and NaCl uptake in the gills of freshwater bivalves might be further monitored. We anticipate that our model can provide the fundamental properties and methodology to portend broad development of commercial and research applications based on the low cost and procedural and conceptual simplicity of these methods.

The proposed gill-based artificial membrane interface can link with measured bivalve data to quantitatively assess the effects of environmental factors on the biouptake kinetics, ion bioavailability, and electrophysiological performance of membrane devices and the variability of bivalve biodynamics and metabolic availability [10, 44-46]. Successful implementation of *in situ* biomonitoring is contingent upon understanding how bioavailability of metals, biological, and electrophysiological factors affect the artificial membrane interface kinetically and dynamically [12, 13, 47, 48]. Additional research concerning the gill architecture and geometry of transfer regions [31, 49-50] and dynamics in electrophysiological performances in clams is still necessary to improve the model.

### 3. Materials and Methods

### 3.1 Integration model

The biologically based kinetic reaction of a metal-ligand process in a membrane interface can be described by the Nernst equation as:

(1) $$\Delta E=\Delta E^{0}-\frac{RT}{nF}\ln\left(\frac{\left\{ML\right\}}{\left[M]\{L\right\}}\right),$$

where Δ*E* and *n* are the measured redox potential (V) as an electromotive force (e.m.f.) and the number of electrons transferred, respectively, Δ*E*^0^ is the standard state potential, *R* is the gas constant (8.3 J mol^-1^ K^-1^); *T* is absolute temperature (°K); [] and {}denote the bulk concentration (μg L^-1^) and free ion concentration of a sensitive site on surface in the organism (mole L^-1^), respectively; *M* and *L* are the metal concentration and ligand in solution, respectively (mole L^-1^). {*L*} in Eq. (1) can be seen as the site of toxic action in the BLM scheme as:

(2) $$\left[M\right]+\left\{L\right\}\overset{K_{S}}{↔}\left\{ML\right\}\overset{K_{\mathrm{int}}}{\to }\left\{M_{\mathrm{int}}\right\}+\left\{L\right\},$$

where *k*_int_ is internalization rate constant (hr^-1^) and {*M*_int_} represents the metal has been internalized with membrane carrier ligands (mole g^-1^).

Generally, the metal transfer across a biological membrane is assumed to be a first-order process. The internalization flux (*J*) can be directly related to any metal species in equilibrium, including gill metal burden {*ML*} as:

(3) $$J=k_{\mathrm{int}}\cdot \left\{ML\right\}$$

We obtained the electrochemistry–based mechanistic model to capture the relationships between internalization flux (uptake) and electrons transferred potential by linking Eqs. (1) and (3),

(4) $$\Delta E=\Delta E^{0}-\frac{RT}{nF}\ln\left(\frac{k_{\mathrm{int}}\cdot \left\{ML\right\}}{k_{\mathrm{int}}\cdot \left[M\right]\cdot \left\{L\right\}}\right)=\Delta E^{0}-\frac{RT}{nF}\ln\left(\frac{J}{k_{\mathrm{int}}\cdot \left[M\right]\cdot \left\{L\right\}}\right)$$

Acute metal toxicity is always associated with inhibition of sites involved in active uptake at gills, resulting in death from failure to maintain homeostasis. We employed the physiological-based mechanistic approach associated with acute metal toxicity to identify species sensitive to metal exposure and further to predict toxic response of biological behavior in *C. fluminea*.

### 3.2 Clam gill-based electrophysiological response model

The importance of metal bioavailability in metal-ligand chemical reactions is best described by Michaelis-Menten (M-M) kinetics. The internalization flux is *J*_max_×[*S*] /(*K*_m_+[*S*]) where [*S*] is metal activity concentration, *J*_max_ is the maximum internalization flux, and *K*_m_ is the M-M affinity constant, representing the metal activity concentration at which the internalization flux equals *J*_max_/2. When [*S*] is abundant, *K*_m_ becomes insignificant; however, when [*S*] is low, *K*_m_ becomes relevant. We have developed a model (called Cu-BLM-*Corbicula* model) [31] to link acute Cu toxicity and its effect on valve closure behavior in freshwater clam *C. fluminea* to support the biotic ligand model (BLM). That model confirms that BLM could be improved to analytically and rigorously describe the bioavailable fraction of metal causing toxicity to valve closure behavior in freshwater *C. fluminea*. We have also provided a flux transport model based on BLM and M-M kinetics to link valve closure behavior and Na transport mechanism in *C. fluminea* [52] (Figure 1B).

Table 3 lists the essential mathematical equations used to describe the Cu-BLM-*Corbicula* model and the flux-biological response framework. Table 3 embraces Na transport-valve closure response model, Na transport, and Cu internalization flux kinetics.

Here we integrated flux-biological response mechanisms and Cu-BLM-*Corbicula* model (Eqs. (T1) – (T3)), taking into account the bioavailability and physiological response, into thermodynamics-based Nernst equation to formulate a clam gill-based electrophysiological response model. We firstly linked electrochemistry–based mechanistic model (Eq. (T4)) and Na transport-valve closure response model (Eqs. (T4) – (T6)) to obtain the key relationships among valve closure response, Na uptake rate, and gill Na membrane potentials:

(5) $$\begin{array}{lll} E_{{\text{Na}}^{+}} & = & \frac{RT}{nF}\ln\left(\frac{J_{{\text{Na}}^{+}}(\phi )}{k_{\mathrm{int}}\;\cdot \;[{\text{BL}}^{-}]\;\cdot \;\left\{{\text{Na}}^{+}\right\}}\right)\\ & = & \frac{RT}{1\;\cdot \;F}\ln\left(\frac{J_{\max}\times \left[1-\frac{1\times \phi^{m(\Delta t)}}{{\left[\text{ER}50_{\phi }(\Delta t)\right]}^{m(\Delta t)}+\phi^{m(\Delta t)}}\right]}{k_{\mathrm{int}}\;\cdot \;[{\text{BL}}^{-}]\;\cdot \;\left\{{\text{Na}}^{+}\right\}}\right), \end{array}$$

where *E*_Na+_ represents the gill Na membrane potential (mV), *ϕ* is a {Cu^2+^}-dependent clam valve closure response function taking into account external Na^+^ activity based on Cu-BLM-*Corbicula* model (Eqs. (T1) – (T3)), *m*(Δ*t*) is the response time-dependent Hill coefficient, ER50*_ϕ_*(Δ*t*) is the 50% effective response due to the % inhibition o Na^+^ uptake rate, and [BL^−^] is the concentration of unoccupated gill BL sites (μmol g^-1^).

We refined Eq. (5) for further predicting the variable membrane potential based on different ion species transporting across gill membrane in *C. fluminea*. We incorporated Na transport kinetics (Eqs. (T7) – (T9)) into Eq. (5) to describe the performance of Na membrane potentials:

(6) $$E_{{\text{Na}}^{+}}=\frac{RT}{1\cdot F}\ln\left(\frac{\frac{J_{{\text{Na}}^{+},\max}\left(\left\{{\text{Cu}}^{2+}\right\}\right)\times \left\{{\text{Na}}^{+}\right\}}{K_{m,{\text{Na}}^{+}}\left(\Delta t,\left\{{\text{Cu}}^{2+}\right\}\right)+\left\{{\text{Na}}^{+}\right\}}}{k_{\mathrm{int}}\cdot \left[{\text{BL}}^{-}\right]\cdot \left\{{\text{Na}}^{+}\right\}}\right)$$

On the other hand, Cu membrane potential (*E*_Cu^2+^_) can be described by the Cu internalization flux kinetics (Eq. (T10)) as:

(7) $$E_{{\text{Cu}}^{2+}}=\frac{RT}{2\cdot F}\ln\left(\frac{\frac{J_{{\text{Cu}}^{2+},\max}\times \left\{{\text{Cu}}^{2+}\right\}}{K_{m,{\text{Cu}}^{2+}}\;+\left\{{\text{Cu}}^{2+}\right\}}}{k_{\mathrm{int}}\cdot \left[{\text{BL}}^{-}\right]\cdot \left\{{\text{Cu}}^{2+}\right\}}\right).$$

Eqs. (6) and (7) provide the information of an accurately electrophysiological response–based mechanisms to estimate the gill membrane potentials for further estimating the waterborne Cu toxicity.

By linking Ussing flux ratio equation [24, 53] with Eq. (6), the external Cu concentration-dependent internal (blood) Na concentration in *C. fluminea* can be estimated to be:

(8) $${\left[{\text{Na}}^{+}\right]}_{i}=\left({\left[{\text{Na}}^{+}\right]}_{o}\frac{J_{\text{o}}^{\text{Diffusion}+\text{Excretion}}}{J_{\text{i}}^{\text{Diffusion}}}\right)\exp\left(\frac{{\text{FE}}_{\text{Na}}^{\text{Active}}}{\text{RT}}\right),$$

where [Na^+^]_i_ and [Na^+^]_o_ are the internal (blood) and external Na concentrations (μM), respectively, the Ussing flux ratio $\left(J_{\text{o}}^{\text{Diffusion}+\text{Excretion}}/J_{\text{i}}^{\text{Diffusion}}\right)$ could be obtained from [24] and was estimated to be 5.74, and $E_{\text{Na}}^{\text{Active}}\;(\text{mV})$ is the Na membrane potential due to the active transport mechanism that can be estimated by our present model framework.

## 5. Conclusions

Our analysis of Cu bioavailability and electrophysiological response interactions in *C. fluminea* leads to several conclusions. We present an ecotoxicologically-electrophysiologically inspired model for the kinetic reconsideration of the clam valve response behavior that incorporates Na active transport mechanism. It entails a highly nonlinear interaction among external Cu bioavailability, Cu-gill ligand binding affinity, Na/Cu internalization kinetics, and depolarization processes of gill transmembrane potentials. The framework captures the features observed in model applications including (*i*) 50% inhibitory Cu^2+^ activities or Na membrane potential and uptake rate are estimated to be 0.072 and 0.043 μM, with a stoichiometry of 3 Cu^2+^: 1 *E*_Na_ and 1*J*_Na_, (*ii*) the external Cu^2+^-dependent internal Na concentration can be parsimoniously estimated, and (*iii*) the site-specific clam gill potentials can be predicted in the aquaculture settings. Our study suggests that a detailed understanding of the nature of ion bioavailability–electrophysiology interactions, together with identification of valve response behaviors validated in an aquaculture setting, can be combined with physiologically-based toxicokinetics and toxicodynamics to identify the sites and mechanisms of action of metabolically available metal and stored detoxified metal in aquaculture species.

## Figures and Tables

**Table 1.** Comparison between published data and our predicted values of clam gill potentials in equilibrium and nonequilibrium conditions.

	Gill (transepithelial) potential (mV)	
	Equilibrium	Nonequilibrium
McCorkle and Dietz [24]	−74 (estimated)	–7 (measured)
This study a	−84.2 (−93.9 − −67.9) b	−8.2 c

a Water chemistry characteristics are based on McCorkle and Dietz [24].

b Calculated by $E_{i}^{\text{Total}}=RT/nF\ln\left(J_{i}^{\text{Total}}/(k_{\mathrm{int}}[BL^{-}]\{{\text{Na}}^{+}\})\right)$ where $J_{i}^{\text{Total}}=J_{i}^{\text{Exchange}}+J_{i}^{\text{Passive}}+J_{i}^{\text{Active}}=0.67J_{i}^{\text{Total}}+0.057J_{i}^{\text{Total}}+0.273J_{i}^{\text{Total}}$ [24] in that parenthesis shows 95% CI.

c $E_{i}^{\text{Active}}=E_{i}^{\text{Total}}-E_{i}^{\text{Diffusion}}$ where $E_{i}^{\text{Diffusion}}=RT/\text{nF}\ln\left(\left(J_{i}^{\text{Exchange}}+J_{i}^{\text{Passive}})/(k_{\mathrm{int}}[BL^{-}]\{{\text{Na}}^{+}\}\right)\right)=-76\text{mV}$ and therefore that $E_{i}^{\text{Active}}=-84.2-\left(-76\right)\text{mV}=-8.2\text{mV}$.

**Table 2.** Measured pH and temperature values with the ion activities of key water chemistry characteristics calculated by WHAM from published data for two selected clam farms of Changhua and Hualien

			Ion activities (mM)				
	pH	Temp. (°C)	Ca^2+^	Mg^2+^	Na^+^	Cl^−^	${\text{SO}}_{4}^{2-}$
Changhua a	8.01±0.19	29.3±0.9	0.41±0.14	0.34±0.08	0.43±0.23	0.40±0.25	0.098±0.15
Hualien a	7.80	30.5	0.36	1.17	12.28	55.57	1.42

a Adopted from Liao et al. [31] where data are represented as mean ± SD (*n* = 3).

**Table 3.** The mathematical descriptions for Cu-BLM-Corbicula model associated with Na transport mechanism and valve closure response in *C. fluminea* in response to waterborne Cu (see text for the meanings of symbols)

Cu-BLM-*Corbicula* model a (T1) $\phi \left(\Delta t,{\text{Cu}}^{2+}\right)=\frac{\phi_{\max}\times {\left\{{\text{Cu}}^{2+}\right\}}^{n\left(\Delta t\right)}}{{\left[\text{EC}50{\left(\Delta t\right)}_{\text{CuBL}}\right]}^{n\left(\Delta t\right)}+{\left\{{\text{Cu}}^{2+}\right\}}^{n\left(\Delta t\right)}}$ (T2) $$\begin{array}{l} \text{Time}-\text{varying Hill coefficient function in valve closure response}\\ n\left(\Delta t\right)=1.221+0.988\exp\left(-\Delta t/37.7\right),r^{2}=0.89 \end{array}$$ (T3) $$\begin{array}{l} \text{Time}‐\text{varying BLM}‐\text{predicated}\;50\%\text{effective response concentration function}\\ \text{EC}50{\left(\Delta t\right)}_{\text{CuBL}}=\frac{f_{\text{CuBL}}^{50\%}\left(\Delta t\right)}{\left(1-f_{\text{CuBL}}^{50\%}\left(\Delta t\right)\right)}\left(\frac{1+K_{\text{CaBL}}\;\left\{{\text{Ca}}^{2+}\right\}+K_{\text{MgBL}}\;\left\{{\text{Mg}}^{2+}\right\}+K_{\text{NaBL}}\;\left\{{\text{Na}}^{+}\right\}+K_{\text{HBL}}\;\left\{{\text{H}}^{+}\right\}}{K_{\text{CuBL}}+K_{\text{CuOHBL}}K_{\text{CuOH}}\;\left\{{\text{OH}}^{-}\right\}+K_{{\text{CuCO}}_{3}\text{BL}}K_{{\text{CuCO}}_{3}}\;\left\{{\text{CO}}_{3}^{2-}\right\}}\right) \end{array}$$ Sodium transport - valve closure response model b (T4) $$\begin{array}{lll} J_{{\text{Na}}^{+}}\;\left(\phi \right) & \equiv & J_{{\text{Na}}^{+}}\;\left(\Delta t,\phi \left(\Delta t,{\text{Cu}}^{2+},{\text{Na}}^{+}\right)\right)=J_{\max}\times \left(1-I_{J_{{\text{Na}}^{+}}}\;\left(\Delta t,\phi \left(\Delta t,{\text{Cu}}^{2+},{\text{Na}}^{+}\right)\right)\right)\\ & = & J_{\max}\times \left[1-\frac{1\times \phi^{m\left(\Delta t\right)}}{{\left[\text{ER}50_{\phi }\left(\Delta t\right)\right]}^{m\left(\Delta t\right)}+\phi^{m\left(\Delta t\right)}}\right] \end{array}$$ (T5) $$\begin{array}{l} \text{Time}‐\text{varying Hill coefficient function in inhibition of}\;{\text{Na}}^{+}\text{uptake}\\ m\left(\Delta t\right)=24.33-778.43/\Delta t,r^{2}=0.97 \end{array}$$ (T6) $$\begin{array}{l} \text{Time}‐\text{varying}50\%\text{effective response function in inhibition of}\;{\text{Na}}^{+}\text{uptake}\\ \text{ER}50_{\phi }\left(\Delta t\right)=84.15-1103.27/\Delta t,r^{2}=0.95 \end{array}$$
Sodium transport kinetics b (T7) $J_{Na^{+}}\left(\Delta t,{\text{Cu}}^{2+},{\text{Na}}^{+}\right)=\frac{J_{\max}\left(\left\{{\text{Cu}}^{2+}\right\}\right)\times \left\{{\text{Na}}^{+}\right\}}{K_{m}\left(\Delta t,\left\{{\text{Cu}}^{2+}\right\}\right)+\left\{{\text{Na}}^{+}\right\}}$ (T8) $$\begin{array}{l} \{{\text{Cu}}^{2+}\}‐\text{dependent maximum}\;{\text{Na}}^{+}\text{uptake function}\\ J_{\max}\left(\left\{{\text{Cu}}^{2+}\right\}\right)=0.345+12.90\exp\left(\frac{-\left\{{\text{Cu}}^{2+}\right\}}{6.154\times 10^{-8}}\right),r^{2}=0.85 \end{array}$$ (T9) $$\begin{array}{l} \text{Response time}‐\text{and}\{{\text{Cu}}^{2+}\}‐\text{dependent half}‐\text{saturation affinity constant function}\\ K_{m}\left(\Delta t,\left\{{\text{Cu}}^{2+}\right\}\right)=a_{1}\left(\Delta t\right){\left\{{\text{Cu}}^{2+}\right\}}^{a_{2}\left(\Delta t\right)}\\ a_{1}\left(\Delta t\right)=3.84+193.66\exp\left(-\Delta t/136.23\right),r^{2}=0.99\\ a_{2}\left(\Delta t\right)=0.862\exp\left(-\Delta t/2875.88\right),r^{2}=0.68 \end{array}$$
Copper internalization flux kinetics b (T10) $J_{{\text{Cu}}^{2+}}\equiv \frac{{\left[\text{CuBL}\right]}_{\text{T}}}{\Delta t}=\frac{J_{{\text{Cu}}^{2+},\max}\times \left\{{\text{Cu}}^{2+}\right\}}{K_{m,{\text{Cu}}^{2+}}+\left\{{\text{Cu}}^{2+}\right\}}$

a Adopted from Liao et al. [31].

b Adopted from Liao et al. [52].
